# Characterizing natural degradation of tetrachloroethene (PCE) using a multidisciplinary approach

**DOI:** 10.1007/s13280-020-01418-5

**Published:** 2020-12-02

**Authors:** Sofia Åkesson, Charlotte J. Sparrenbom, Catherine J. Paul, Robin Jansson, Henry Holmstrand

**Affiliations:** 1 Department of Geology, Lund University, Sölvegatan 12, 223 62 Lund, Sweden; 2 Applied Microbiology, Department of Chemistry, Lund University, P.O. Box 124, 221 00 Lund, Sweden; 3 Water Resources Engineering, Department of Building and Environmental Technology, Lund University, P.O. Box 118, 221 00 Lund, Sweden; 4 Department of Environmental Science (ACES), Stockholm University, Svante Arrhenius väg 8, 106 91 Stockholm, Sweden

**Keywords:** Chlorinated solvents, Compound-Specific Isotope Analysis, Geophysics, Groundwater, Natural degradation, Quantitative Polymerase Chain Reaction

## Abstract

A site in mid-western Sweden contaminated with chlorinated solvents originating from a previous dry cleaning facility, was investigated using conventional groundwater analysis combined with compound-specific isotope data of carbon, microbial DNA analysis, and geoelectrical tomography techniques. We show the value of this multidisciplinary approach, as the different results supported each interpretation, and show where natural degradation occurs at the site. The zone where natural degradation occurred was identified in the transition between two geological units, where the change in hydraulic conductivity may have facilitated biofilm formation and microbial activity. This observation was confirmed by all methods and the examination of the impact of geological conditions on the biotransformation process was facilitated by the unique combination of the applied methods. There is thus significant benefit from deploying an extended array of methods for these investigations, with the potential to reduce costs involved in remediation of contaminated sediment and groundwater.

**Electronic supplementary material:**

The online version of this article (10.1007/s13280-020-01418-5) contains supplementary material, which is available to authorized users.

## Introduction

Chlorinated solvents are common contaminants of soil and groundwater at numerous sites of active and historical dry cleaning facilities and industrial operations. Presently, nearly 3000 sites are known or suspected to be contaminated with chlorinated solvents in Sweden alone (SEPA 2015). Chlorinated solvents are dense non-aqueous phase liquids (DNAPLs), and this character gives them a special dispersal pattern and complex distribution that is controlled by local geological settings (Pankow and Cherry 1996). As these substances are harmful, and have been proved or are considered to be carcinogens (IARC 2014), sites contaminated with these solvents are highly prioritized for remediation. In situ remediation is recommended by the Swedish EPA as a more sustainable environmental strategy (SEPA 2014) than the traditional “dig and dump” treatment of contaminated sediments. However, in order to monitor and optimize this remediation, a reliable investigation methodology to validate the degradation progress is required.

Complete biodegradation of PCE occurs in several ways, either by *Dehalococcoides mccartyi* alone or by a bacterial consortium where different bacteria carry out one or more steps of biodegradation (Stroo et al. 2013). *D. mccartyi* is the only bacterium known to be able to completely degrade PCE to ethene (He et al. 2003). The slightly stronger molecular bond and lower reaction rate of ^13^C-Cl, compared to that of ^12^C-Cl, leads to enrichment of ^13^C in the remaining molecular PCE pool during degradation. Microbial biotransformation is considered to have the largest effect on this isotope composition, whereas abiotic processes tend to impose smaller shifts of less significance (Hunkeler et al. 2008). The use of direct current resistivity and induced polarization (DCIP) tomography to investigate the contaminated area gives a continuous spatial model of the subsurface based on its different physical response to an electrical current (e.g., Cardarelli and Di Filippo 2009; Power et al. 2014; Johansson et al. 2016; Sparrenbom et al. 2017). As microbial consortia are negatively charged (Abdel Aal et al. 2006; Atekwana and Slater 2009) and gas is produced as a co-product in degradation, DCIP has previously been shown to be able to monitor gas migration (Rosqvist et al. 2011; Auken et al. 2014).

We present here an investigation of a site that is contaminated by chlorinated solvents (tetrachloroethene [PCE] and its metabolites), where only natural attenuation has occurred. The site is located in Hagfors in mid-western Sweden and hosted the largest dry cleaning facility in Sweden. It was operational until the 1990s and resulting spills have led to significant subsurface contamination of the soil and groundwater by PCE and its metabolites (trichloroethene, *cis*-dichloroethene, and vinyl chloride). To understand and characterize the subsurface biogeochemical system, we have used a multidisciplinary approach including analysis of concentrations of contaminants in the groundwater, including major and minor ions; DNA-based quantification and characterization of the bacteria present at the site; Compound-Specific Isotope Analysis (CSIA) focusing on *δ*^13^C in PCE; and DCIP tomography. We have used methods to detect and quantitate both *D. mccartyi* and total amounts of bacteria using detection of the 16S rRNA gene to characterize the biodegradation taking place. DNA-based methods assessing the biological degradation are complemented by CSIA to quantify the degradation state of PCE based on the shift in the molecular carbon isotope composition concomitant to the degradation process (Elsner et al. 2005). We investigate whether the indications of microbial activity correlate with the state of degradation, obtained from both groundwater chemistry and CSIA, and investigate the possibility to apply DCIP as a diagnostic method for assessing the geo-spatial PCE degradation state. The concentrations of chlorinated solvents have been previously observed to correlate to information from CSIA by, for example, Hunkler et al. (1999), and microbiological phenomena by, for example, Lu et al. (2006). Natural remediation and changing concentrations of the PCE and metabolites have also been complemented with CSIA and microbiology observations by Fletcher et al. (2011), Damgaard et al. (2013), and Kuder et al. (2013). The link between DCIP results and chlorinated solvents/DNAPLs has been described by Ajo-Franklin et al. (2006), Champers et al. (2010), and Johansson et al. (2019). Microbiological observations have been linked to DCIP signals by Davis et al. (2006), Atekwana and Slater (2009), and Abdel Aal et al. (2010) and others. Thus, while previous studies have used some of these methods in combination, they have never been used together. The objectives of this study were to validate ongoing degradation by natural processes, and investigate the value of using the applied array of methods for monitoring of these processes.

### Study area

**Fig. 1:**
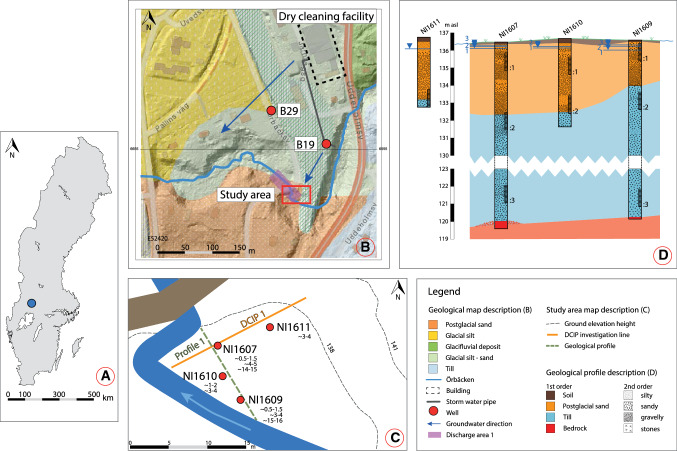
**a**–**d** Location and geological map of the field site. **a** Site location (blue dot) in Sweden. **b** Overview of the site, showing the surface geology and elevation as hill shape. External information has been incorporated with a map from ©SGU (http://www.sgu.se/sgu/sv/produkter-tjanster/kartvisare/index.html). The green grid shows the filling material of the partly anthropogenic plateau. **c** Close up of the outline of the study area at the point bar including well names, filter depth as m below surface, DCIP investigation line, and a geological profile shown in **d**. **d** Geological profile for the cross section indicated by the green line in **c**. Filter screens are marked in each well as a linear raster including names. The geological profiles are made from bore logs (Larsson et al. 2017) and simplified with color coding. Groundwater levels were measured during the fieldwork in this study

The Hagfors dry cleaning site (Fig. 1a) served the military during 1970–1979, with continuous operations until 1993 when it was taken over by the county council (Nilsen 2013). This long period of PCE usage with a known but unquantified loss of product has resulted in a highly contaminated site (Nilsen 2013). There are two source areas: a primary area beneath the building, with a plume following the groundwater flow direction (northern arrow in Fig. 1b); and a secondary area extending from a sewer system from the building and leading out to a small stream. It is unclear if the sewer system transported the contamination directly or supported transport along the ballast filling in the duct ditch. The highest concentration of contaminants at the site is found around well B19 in the secondary source area (Fig. 1b), where PCE has been found in free phase (Larsson et al. 2017). The secondary source plume extends towards a point bar in the stream and down into a ravine. This investigation was focused on that point bar where the plume discharges into the stream. Calculations within “discharge area 1” (Fig. 1b) estimated a PCE discharge of 59 kg year^−1^ (Nicklas Larsson pers. comm.).

The Hagfors site is located below the highest shoreline from the latest glaciation, which has generated extremely complex geology in this area. Natural sediments at the site consist of both glacial and post-glacial deposits, and anthropogenic material (Fig. 1b). The building that accommodated the dry cleaning facility is located on a former railroad yard and consists of an artificial embankment plateau of unknown construction and ballast material. Filling material is marked as a grid on the geological map (Fig. 1b) with the background color coding showing the natural deposit beneath. The detailed study area consists of a point bar, with three geological units existing above the bedrock. Starting from the bottom, a compact silty till is overlain by a sand unit containing isolated lenses of silt and gravel, with a transition unit of gravely sand between the two (Fig. 1d) (Larsson et al. 2017). The investigation area thus forms two hydrogeological units, one unconfined in the top and one semiconfined beneath, with a higher hydraulic pressure. The unconfined top sand is interpreted to have a high hydraulic conductivity and the lower till unit seems to act as an aquitard within the system. Four Sonic-drilled monitoring wells are installed (Fig. 1c) with one, two, or three 1-m filter screens in the point bar (Table 1) (Larsson et al. 2017). Additionally, two reference wells at the embankment plateau (i.e., B19 and B29) have been sampled. The stream is partly meandering and partly forced into its groove; it flows via a culvert under the tail of the plateau. From an older map (1928) it is known that the stream previously had a more meandering groove (Larsson et al. 2017). Most likely it has meandered since it was formed, leaving paleo channels that are today filled with sediments.

**Table 1:** Investigated filter screens including depth and corresponding geological unit

Well name	Numbers of filter screens	Filter screens ID	Filter screen depth (m below surface)	Geological unit
NI1607	3	NI1607:1	0.61–1.61	Sand
		NI1607:2	3.99–4.99	Top till
		NI1607:3	14.43–15.43	Bottom till
NI1609	3	NI1609:1	0.18–1.18	Sand
		NI1609:2	2.9–3.9	Top till
		NI1609:3	14.91–15.91	Bottom till
NI1610	2	NI1610:1	1.05–2.05	Sand
		NI1610:2	3.23–4.23	Bottom sand/transition into till
NI1611	1	NI1611	1.36–2.36?	Sand/transition into till *
B19	7	B19 (deepest)	22.07–22.17	Gravel
B29	1	B29	14.5–15.5	?

*Uncertain about filter depth location. Later slug test suggested mainly sand (Nikolas Benavides Höglund, pers. comm.)

## Materials and methods

### Data collection and analysis

The field campaign took place in late April 2017. All water samples at the point bar were taken with an Eijkelkap Peristaltic Pump, while B29 was purged with a Solinst Bladder Pump and B19 using a Waterra Pump. The change in pumps was due to depth and diameter of wells. The sampling procedure was to pre-purge each well and monitor physical properties (i.e. temperature, oxidation–reduction potential [ORP], pH, electrical conductivity, total dissolved solids [TDS], and salinity) with an Aquaread Aquaprobe® AP-800. When stable parameters were obtained, the samples for analysis were taken in the following order: CSIA, physical and chemical samples, and microbiological samples. Eleven filter screens in total were sampled for this study. DCIP was measured during the same field campaign by Jansson (2018) and is described in more detail in Sect. 2.1.3.

The groundwater for CSIA was collected in 1 L amber glass flasks with PTFE lined screw caps (Hunkeler et al. 2008). The pump rate was kept low to avoid cavitation and minimize degassing of contaminants due to a reduced water pressure. The sample was allowed to overflow briefly, before the addition of 10 mL of HCl (19%) as preservative. The CSIA samples were sent to Isodetect GmbH, Leipzig, for analysis of the carbon isotope composition using continuous flow methodology (Hunkler et al. 2008). The groundwater samples for physical and chemical analysis were collected in five bottles: two amber glass bottles (100 mL), containing conservation liquid for chlorinated solvents concentrations; two plastic (PEHD) bottles of 150 mL for metals; and one 500 mL plastic (PEHD) bottle for the remaining analysis. ALcontrol AB performed the physical and chemical analysis. Microbial cells for DNA extraction were collected by filtering water through 0.2 µm Isopore filters in a filter-holding funnel on top of an Erlenmeyer suction flask under vacuum obtained by peristaltic pump. The filtering continued until the filter paper was clogged, for three replicates at each sampling point and the volume was noted for each. For well B29, neither of the filter papers clogged, and the filtering process stopped after 2 L water had been filtered. The papers were placed in Petri dishes (glass), which were directly put on dry ice. They were kept as cold as possible with help of ice clamps during transport to the lab, where they were frozen at − 20  °C until DNA extraction.

#### CSIA analysis

CSIA analysis quantifies the molecular carbon isotope composition, where a shift in the ratio between ^13^C and ^12^C is associated to biodegradation. An automated purge-and-trap system was used to extract the target molecules from the groundwater, followed by gas-chromatography isotope-ratio mass spectrometry (GC-IRMS), with inline combustion conversion to CO_2_. Authentic standards were used to correct for any analytical artifacts in the obtained isotope data. The results are reported as

$$\delta^{13} {\text{C}} = \left( {R_{s} /R_{r} } \right) - 1,$$

where *R*_s_ is the ^13^C/^12^C ratio of the sample and *R*_r_ is the ^13^C/^12^C ratio of the Vienna Pee Dee Belemnite reference material (V-PDB) (Coplen 2011). The biodegraded fraction (*B*) of PCE is calculated as

$$B = 1 - F = 1 - \left( {1000 + \delta ^{{13}} C_{S} /1000 + \delta ^{{13}} C_{0} } \right)^{{1000/\varepsilon }}$$

where *F* is the degraded fraction of PCE, and *δ*^13^C_S_ and *δ*^13^C_0_ are the observed isotope compositions in the sample and the initial isotope composition of the pristine contaminant, respectively. Degradation of PCE is assumed to consistently enrich the molecular pool in ^13^C. The sample with the lowest *δ*^13^C was therefore taken as representative of the initial isotope composition, with due consideration to source-zone proximity and influence of physical processes such as isotope fractionation during evaporation of PCE to the atmosphere. The isotope enrichment factor, *ε*, is obtained from the literature. The dehalogenative reduction of PCE has been found to yield *ε* values in the range − 2.0 to − 5.5‰ (Elsner et al. 2005; Wiegert et al. 2013). We have chosen the value − 5.5‰ since it gives more conservative estimates for the biodegraded fraction, thus reducing the risk of overestimating the degradation state.

#### Quantification of total bacteria and *D. mccartyi* by quantitative PCR (qPCR)

Quantitative PCR is a molecular DNA-based method that allows the number of gene copies for a specified target to be determined in a sample. While it can be highly specific for individual species, the assay can also be designed to capture more general DNA targets, such as the 16SrRNA gene shared by all bacteria. It is highly precise, but as it is a DNA-based method, it cannot distinguish between bacteria (in this study) that are living or dead (Smith and Osborn 2009). In order to obtain DNA for the qPCR reaction, the filter paper containing cells collected from the largest volume of filtered water from each filter screen was chosen for DNA extraction, except B29, where no filter completely clogged and the most clogged was used. DNA was extracted using the FastDNA™ Spin Kit for soil according to manufacturer’s instruction. Primers and probes were as described in Nadkarni et al. (2002) for quantification of total amounts of bacteria, and He et al. (2003), for *D. mccartyi.* Chromosomal DNA from *Escherichia coli* DSM1116 was used as the positive control for all bacteria and the linearized plasmid, pBAV1 (provided by Frank Löffler, University of Tennessee) as the specific positive control for *D. mccartyi*. To reduce inhibition from organic matter, all DNA templates from environmental samples were diluted 1:5 in MilliQ water. DNA concentrations in the standard dilution series were measured using ThermoFisher Qubit Florescence 3.0. Thermocycling conditions for quantification of total bacteria followed Nadkarni et al. (2002) and Matturro et al. (2013) for *D. mccartyi*. For general bacteria, the qPCR was performed in 20 μL reaction volumes containing 5 μL of template DNA, 1X ExTaq Buffer (TaKaRa), 0.2 mM dNTPs, 2 mM MgCl_2_, 0.3 μM of each primer, 0.2 μM TaqMan DNA probe, 0.1 mg mL^−1^ bovine serum albumin (BSA), and 0.05 U μL^−1^ ExTaq HS. qPCR was performed in a thermal cycler (Roche LightCycler 2.0) using the following program: an initial denaturation step at 95 °C for 15 min followed by 45 cycles of denaturation at 95 °C for 10 s, annealing at 60 °C for 20 s and elongation at 72 °C for 30 s. For detection of the BAV1 gene in *D. mccartyi*, qPCR was performed in 20 μL reaction volumes containing 2 μL of template DNA, 1X ExTaq Buffer (TaKaRa), 0.2 mM dNTPs, 2 mM MgCl_2_, 0.3 μM of each primer, 0.2 μM Dhc1240Probe, 0.1 mg mL^−1^ BSA and 0.05 U μL^−1^ ExTaq HS. qPCR was performed with the same machine as in previous setup. The following program was used: a first initial denaturation step at 50 °C for 2 min and a second initial denaturation step at 94 °C for 10 min, followed by 45 cycles of denaturation at 95 °C for 15 s, annealing at 60 °C for 30 s, and elongation at 72 °C for 30 s.

#### DCIP data acquiring and processing

DCIP data were acquired using an ABEM Terrameter LS2 instrument, which has a fully integrated data acquisition system for measuring resistivity and IP (see also Jansson 2018). Two cables were used, in total 41 electrodes, with 0.5 m spacing between the electrodes with a pole-dipole electrode configuration. The design aimed to obtain high spatial resolution descriptions of the shallow subsurface. Longer electrode layouts were not possible, due to geometry of the point bar area with the known drawback of a limitation in penetration depth, to about 6 m b.g.s. The instrument was set to record data with a sample rate of 3750 Hz and pulse length of 2 s. The measurement was stacked two times to acquire good quality data, and performed with 100% duty cycle according to Olsson et al. (2015). The field conditions in Hagfors adjacent to Örbäcken are ideal for DCIP measurements, with nearly saturated sand and a groundwater table near the ground surface, which assures a low resistance between electrodes and the ground. An electrode contact test was conducted before data acquisition. The position of each electrode and well was determined using a global A Global Navigation Satellite System (GNSS).

Data were processed in the Aarhus Workbench (v. 5.5.0.0) software package and the inversions were performed with Aarhus inversion code: Constant Phase parametrization (Fiandaca et al. 2012). The inversion used L2 norm, as a 20 layered model with medium constraints in the lateral direction (STD: 1.3) and vertical directions (STD: 2). A constrain value of 1.1 means that model parameters were allowed to vary 10% between neighboring cells (Jansson 2018).

## Results

The complete results describing groundwater chemistry, isotopic data, and quantification of bacteria are listed in Table S1.

### Groundwater conditions

**Fig. 2:**
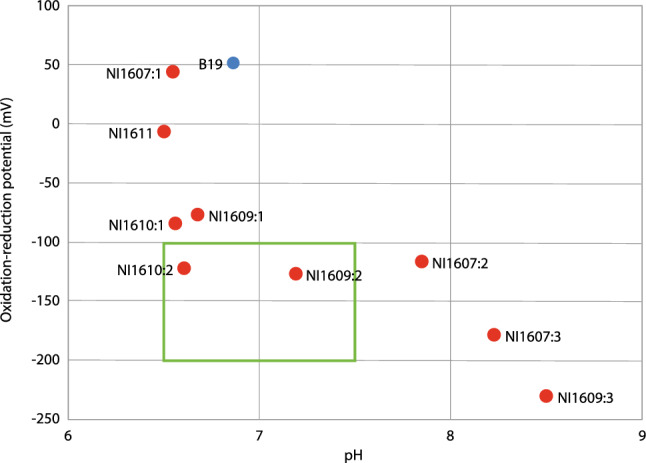
The correlation between pH and ORP shows that the highest pH is found where ORP value is the lowest, and vice versa. The highest pH and lowest ORP values are found in the bottom till (samples ending with “:3”), and in the top till (NI1607:2 and NI1609:2) ORP values are between − 115.3 and − 126.5 mV and pH varies within 6.6–7.85. For the filters in the sand unit, the pH is between 6.5 and 6.67 but the ORP value varies between − 82.8 and 45.1 mV. The analyses result from B19 is marked in blue, since it is not located at the point bar. The green box marks out the optimal conditions for degradation according to AFCEE (2004)

Measured groundwater pressures showed the highest levels in the bottom filter screens and lower levels in the higher filters (Fig. 1d). This verified the described interpretation of the hydrogeological system with a lower aquitard and an aquifer in the sand on top. The connections of groundwater heads between filter screens located in the same formation were ambiguous, due to heterogeneity in the geology. The groundwater temperature increased by depth and ranged from 2.9 to 4.0 °C within the sand, 4.1–4.8 °C in the top till/transition unit, and temperature of 5.9–6.1 °C in the bottom of the till. This was expected since the fieldwork measurements and sampling took place at the end of the cold period of the year. The groundwater parameters measured in the field showed that the different groundwater wells can be divided into three groups based on pH and ORP values as illustrated in Fig. 2. The highest pH values were in the bottom till, with lower values within the top till and transition unit and lowest pH values in the sand unit. The ORP decreased vertically from top to bottom in each well with multiple filter screens; however, the values did not correlate horizontally. pH was the highest where the lowest ORP value was found, with lower pH when the ORP values were higher (Fig. 2). There seems to be a threshold of ORP values between − 100 and − 150 mV, where pH changed between c. 6.5 and almost 8.

The water from filter screens NI1607:2 and NI1609:3 had similarly deviating chemical composition compared to the rest of the samples from the point bar. These screens had higher concentrations of fluoride, chloride, sodium, boron, and lower concentrations of iron, which explains the higher field-parameter values of conductivity, TDS, and salinity. As the water composition from these two filter screens differed significantly from the rest of the water samples, correlation tests were made both with and without data from NI1607:2 and NI1609:3. While they were included the correlations examined by liner regression improved, for example the correlation between sulfate and sodium improved from *r*^2^ = 0.30 to *r*^2^ = 0.93 when these two samples were included.

### Contamination conditions

**Fig. 3:**
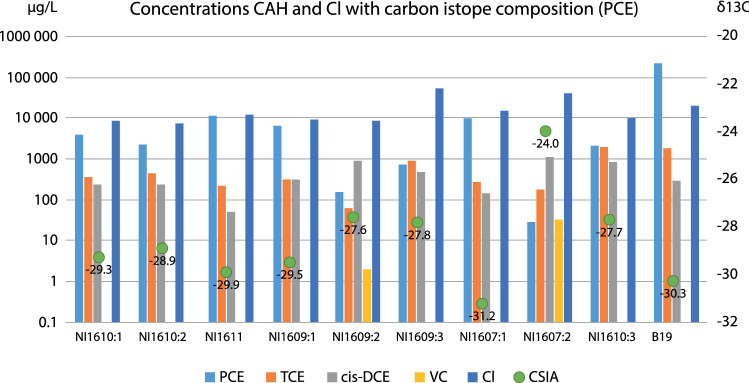
Groundwater data with concentrations of chloride and CAH, with the exception of only including *cis*-DCE. At the right *y*-axis *δ*^13^C-scale and *δ*^13^C values, PCE marked as a green dot and labeled with measurement values. Higher *δ*^13^C is found where vinyl chloride is detected (i.e., NI1609:2 and NI1607:2) in combination with lower concentrations of PCE. NB, logarithmic scale at left *y*-axis

The concentrations of PCE in the samples from the point bar varied between 29 and 11 000 µg L^−1^, where the highest concentrations were found in NI1611, followed by NI1607:1. Lowest concentrations were found in the water taken from the two filters in the top part of the till (NI1607:2 and NI1609:2), which were also the only two samples where vinyl chloride was detected (33 and 2 µg L^−1^, respectively). All samples from the point bar showed degradation down to *cis*-DCE stage, and *trans*-DCE was also found in two samples. For the samples from NI1607:2 and NI1609:3, the concentrations of TCE were higher than for PCE. In NI1607:3 and NI1609:2, the difference in ratio between PCE:TCE was smaller than for the rest of the samples. Contaminant concentrations are presented in Table 2 and Fig. 3. The sample from well B19 had the highest absolute concentration of PCE (220 000 µg L^−1^, Table 2), as expected from previous investigations at the site (Nilsen 2013; Larsson et al. 2017). This concentration suggests the possibly that at this location, PCE may exist in free phase. B19 had increased detection limits for the degradation products due to high concentration of PCE. Well B29 was considered as the “natural background sampling point” and well B19 represented the secondary source zone. However, B29 was not uncontaminated with respect to PCE and metabolites, possibly showing influence from the primary source plume.

**Table 2:** Results from chemical analysis of chlorinated solvents (CAH)

Filter screen	PCE	TCE	*cis*-DCE	*trans*-DCE	1,1-DCE	VC	Ratio PCE:TCE
	μg L^−1^	μg L^−1^	μg L^−1^	μg L^−1^	μg L^−1^	μg L^−1^	
NI1610:1	4000	350	230	< 5.0	< 5.0	< 10	80:7
NI1610:2	2300	450	240	< 5.0	< 5.0	< 10	46:9
NI1611	**11 000**	220	51	< 10	< 10	< 20	50:1
NI1609:1	6600	320	310	< 10	< 10	< 20	165:8
NI1609:2	160	64	890	**2.1**	< 1.0	**2**	5:2
NI1609:3	720	930	470	**2.8**	< 1.0	< 2	**24:31**
NI1607:1	10 000	280	140	< 10	< 10	< 20	250:7
NI1607:2	29	180	1100	< 5.0	< 5.0	**33**	**29:180**
NI1607:3	2100	1900	820	< 5.0	< 5.0	< 10	21:19
B29	39	0.32	**< 0.1**	< 0.1	< 0.1	< 0.2	n.a.
B19	**220 000**	1800	290	< 100	140	< 200	1100:9

Concentrations discussed in the text are indicated in bold

### Degradation state from isotopic analysis

The calculation of the biodegradation (B) requires an isotopic starting point, *δ*^13^C_0_. Sample NI1607:1 had the most depleted *δ*^13^C value (− 31.2‰) and therefore might be taken as representative of the initial isotope composition (*B* = 0). However, evaporation of PCE can induce depletion in ^13^C due to the reduction in molar volume, and increase in vapor pressure, of the ^13^C isotopologue (Huang et al. 1999). Thus, the shallow filter depth (0.6–1.6 m below ground level), combined with the permeable sand at this point, could potentially influence the observed *δ*^13^C. This was supported by isotopically heavier *δ*^13^C values obtained at greater depth in NI1607 (− 24.0 to − 27.7‰) and appears to be a general feature of in the CSIA data set (wells NI1609 and NI1610 follow the same pattern). As the source-zone sample (B19) had a *δ*^13^C value of − 30.3‰ and was obtained from > 22 m depth, with a very high concentration of PCE (~ 220 000 µg L^−1^), this sample was therefore used to define *δ*^13^C_0_ as it was unlikely to have been affected by evaporation of PCE, or extensive biodegradation due to the toxicity of the PCE.

Samples from four out of nine filter screens on the point bar had an isotope shift (*δ*^13^C_0 _– *δ*^13^C) larger than 2‰, indicating significant biodegradation (*B* = 0.37 to 0.69, Fig. S1) (Hunkler et al. 2008). The remaining five filter screen samples also exhibited moderate degradation signatures (*B* = 0.07 to 0.23) and the full data set was as expected, with the decreased PCE concentrations and increasing *δ*^13^C values expected as signatures of microbial degradation of PCE (Fig. S2). The CSIA results showed similar trends to those observed for the concentrations of chlorinated solvents: all groundwater samples with higher concentration of the metabolite (TCE and/or *cis*-DCE) than PCE had a *δ*^13^C value higher than − 28‰ (Fig. 3). This was also observed for NI1607:3 although the concentration of PCE was higher than both TCE and *cis*-DCE.

### Quantitation of the microbial populations

Based on calculations to determine the copy number of the bacterial gene for 16S rRNA, and assuming that this quantitation is proportional to the total bacteria in a sample, the water sample that contained the most bacterial DNA per liter of water was NI1609:1, followed by NI1607:2 and NI1610:2. The fewest bacteria were found in the water sample from the reference well B29, followed by NI1609:3 and NI1610:1, both located at the point bar (Table 3).

**Table 3:** Concentration of bacteria, based on copy numbers of the 16S rRNA gene per mL of filtered water. The results are listed in order of concentration of bacteria detected. Detection of BAV1 from gel electrophoresis is marked as +

Well	Copies per mL filtered water	*D. mccartyi* detected
NI1609:1	3168	
NI1607:2	1647	+
NI1610:2	1551	
NI1611	611	
NI1609:2	602	
NI1607:3	530	+
NI1607:1	237	
B19	206	+
NI1610:1	183	
NI1609:3	63	
B29	6	+

No qPCR product was detected during cycling to indicate the presence of *D. mccartyi* in any sample. When qPCR reaction products from all samples were inspected by gel electrophoresis, a band corresponding to the correct size of amplified DNA (BAV1) was detected in four samples: NI1607:2, NI1607:3, B19, and B29 (Table 3 and Fig. S3). The band with the strongest intensity belonged to NI1607:2. Some larger amplicons were detected in the samples where the correct sized amplicon for the BAV1 product was also detected. This suggests that *D. mccartyi* was present in the samples where the band was visualized but could not be quantitated by qPCR due to the interference and amplification of non-specific products.

### Geoelectrical subsurface conditions

Since the geology of the narrow study area is well investigated and known, the discovered anomalies in the inverted DCIP results are possible to attribute to the occurring contaminants and microbial activities. The expected results of resistivity would be two distinct boundaries, one between the unsaturated–saturated sand and the second between the saturated sand and the silty till, due to their differences in resistivity (Palacky 1987). For the IP-response, we expected an influence from the contamination or microbial activity, or both (e.g. Atekwana and Slater 2009; Johansson et al. 2015).

**Fig. 4:**
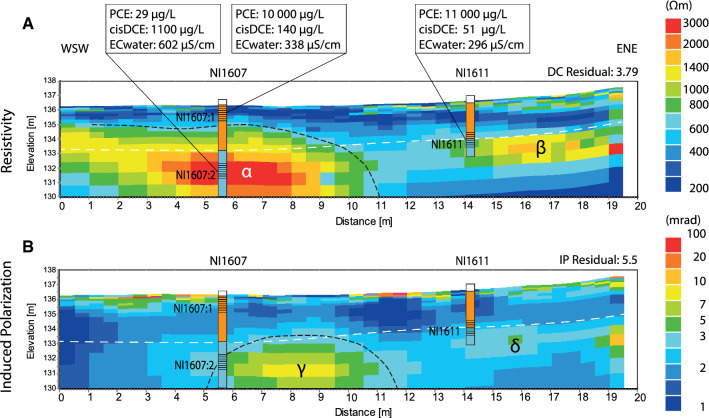
DCIP data from Line 1 passing monitoring well NI1607 and NI1611. **a** Results from the resistivity inverse model with *α* and *β* marking the anomalous areas within the profile. The *α*-anomaly is interpreted to be caused by microbial degradation producing gas (e.g. vinyl chloride and methane) that interfering with current transport. The *β*-anomlay is interpreted as the geological units changing from sand into till with higher resistivity. The low resistive area in between is interpreted as a sand-filled paleochannel. **b** Induced polarization of the same profile shows two anomalies. The *γ-*anomaly is interpreted to be the result of the biodegradation taking place and producing gas within the area, and corresponds to *α* in a, whereas the area of *δ* do not show any major changes

For the resistivity inverted model (Fig. 4a), low resistivity was found in the upper part of the subsurface; however, it varied and was higher in the east north-east surface part of the profile, where there was also more vegetation and litter present. Two anomalies with higher resistivity are marked in Fig. 4a, as *α* and *β*, where *α* is located in the same area as filter screen NI1607:2 and *β* is close by NI1611. The *α* has higher resistivity value than *β*. Groundwater samples taken above and within *α*, i.e., from filter screens NI1607:1 and NI1607:2, showed differences in physical (e.g., EC) and chemical properties (e.g., PCE and metabolites). Within the *α*-anomaly, the groundwater had a higher *cis*-DCE concentration than PCE indicating degradation, while the opposite was observed for the sample taken from the filter screen above the anomaly. Electrical conductivity within the water differed from 338 µS cm^−1^ in NI1607:1 above the *α*-anomaly to 602 µS cm^−1^ in NI1607:2 within the *α*-anomaly. This contradicts with the high resistivity in *α* and high electrical conductivity within the water; however, it could be a result of gas in the pore system blocking transfer of electrical current. The high resistivity anomaly could also correspond to a boulder, although these were not found during drilling of the well NI1607 and, therefore, gas from degradation is the more likely cause of the resistivity anomaly.

The *β*-anomaly did not show as high resistivity compared to *α*-anomaly, and appeared at the same depth as the lithological transition from sand to till. The degradation within NI1611 was not very prominent in the area, suggesting that the *β*-anomaly shows geological changes. This indicates that part of the high resistivity in *α* can also be explained by geological change. The low resistive area between the high anomalies could be a paleochannel in the till formed by the stream’s previous meandering groove, and filled with coarser sediment or more loosely packed material, resulting in a lower resistivity than the surrounding till.

The induced polarization inverted model results (Fig. 4b) showed one larger anomaly (*γ*) in the top part of the till, which was more chargeable than the surrounding area. This *γ*-anomaly could have several explanations, with the most likely being the combination of increased polar metabolites from PCE, an increase of microbial cells, and production of gas, which would all influence the accumulation of charges due to degradation. Each of these factors has been shown to individually contribute to enhancing charging properties (Atekwana and Slater 2009; Kessouri et al. 2019). Other possible explanations could be biologically controlled ion precipitation (Atekwana and Slater 2009), higher content of organic material (Atekwana and Slater 2009; Kessouri et al. 2019), or a higher clay content (Slater and Lesmes 2002) within the till, although clay and organic material were not detected within the till during drilling. The first explanation also correlates well with the microbial results (Table 3), and harmonizes with the interpretation of gas causing the higher resistivity in the *α*-anomaly shown in Fig. 4a. The differences in position between *α* and *γ* could depend on transport of gas downstream of the area, where most production occurs, or roughness of the DCIP data measurements and processing in a coarse mesh, as well as 3D effects. Notable, an anomaly *δ* (located close to the position of the *β*-anomaly) was faintly more chargeable than the surroundings. Since low degradation rates have been found in the *δ*-anomaly area, and this could also indicate that the *β*-anomaly belongs to the geological change.

## Discussion

The objectives for this study were to observe and validate ongoing natural degradation and to show the value of applying the array of methods described for conducting these observations. The combined results from all the different methods clearly describe an area with ongoing microbial degradation. Specifically, the area located near filter screen NI1607:2 (*α* and *γ* in Fig. 4) shows high degradation activity.

The conditions at the study site, with respect to ORP, pH, and dissolved organic carbon (DOC), are not thought to be optimal for microbial degradation of PCE and its metabolites (AFCEE 2004). However, this does not rule out the possibility that some degradation can be occurring, albeit suboptimally, and detection of vinyl chloride supports that conditions could support some degree of microbial degradation beyond DCE. The groundwater from NI1609:2 had ORP and pH within an optimal range, but DOC concentrations were too low to be optimal. For NI1607:2, the pH was slightly too high to be optimal, but it still had conditions that would be able to support slower degradation (AFCEE 2004).

**Fig. 5:**
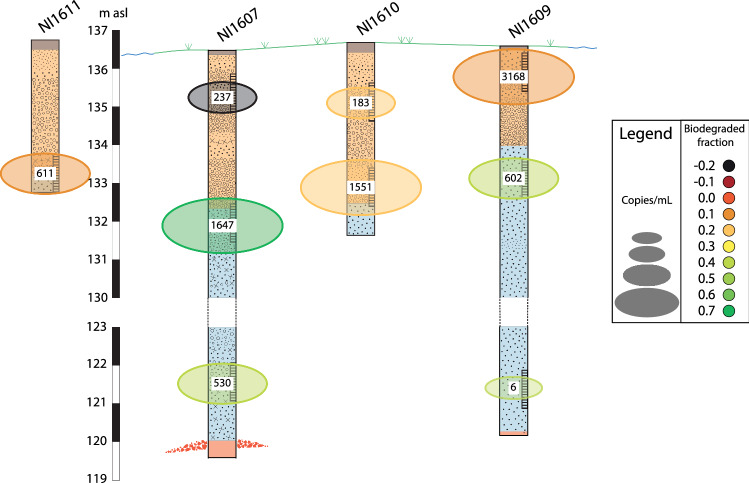
Total amount of bacteria and biodegraded fraction (CSIA) in the geological profile. The highest amount of bacteria and the highest biodegraded fractions are both from the samples of NI1607:2. In the other samples, the two methods do not harmonize. For geological description see Fig. 1d

Bacteria were detected in various amounts in the samples. In general, the most bacteria were found in the samples taken from filters extending from the top of the till into the sand unit, and fewer were observed from the top sand and bottom of the till (Fig. 5 and Table 3). An exception to this was NI1609:1, where we found the most bacteria; however, this filter screen was partly above the groundwater level. Jansson (2018) established top-soil profiles at the point bar. The soil sample closest to NI1609 (S2) showed a transition in color from black to rust going downwards. This indicates fluctuating groundwater levels over time and suggests fluctuations in aerobic and anaerobic conditions, which would be less favorable for the anaerobic bacteria known to degrade chlorinated solvents. The high amount of DNA in the NI1609:2 sample, without clear detection of *D. mccartyi,* reflects that there are plentiful bacteria in this environment. While qPCR for *D. mccartyi* was not able to detect specific products indicating the presence of BAV1 in any samples, gel electrophoresis visualized products of the expected size. This indicated that while BAV1 was present in some of the samples, the conditions in the qPCR assay were not optimal for quantitating this specific target in this study. The protocol that was followed was that described by Matturro et al. (2013) and only modified to adapt to local laboratory routines. However, inhibition of the qPCR reaction, or other interactions of the qPCR matrix, can cause non-specific binding, and could explain both the appearance of the additional bands in the gel electrophoresis analysis and the inability to quantitate the BAV1 product, although it could be visualized. Inhibitors present in DNA extracted from environmental samples can affect amplification and also attenuate fluorescence, impacting detection and accurate quantification (Sidstedt et al. 2019). An impact of environmental inhibitors on detection using this particular qPCR approach has also been reported by others although the number of samples affected, and/or reason for the inhibition was not reported (Clark et al. 2018). Munro et al. (2017) showed a correlation between high numbers of all bacteria and the presence of specific dehalogenating bacteria. Since in the current study we have only looked for *D. mccartyi*, it is possible that other dehalogenating bacteria are conducting the degradation observed at this site, and are included in the total bacterial quantification. In two of the samples, *trans*-DCE was detected, and since this is not a reaction performed by *D. mccartyi* this also supports the hypothesis that other bacteria are performing degradation of PCE and that bacteria able to carry out partial bioremediation of PCE are present (Stroo et al. 2013). Cooperative bacterial interactions supporting biodegradation of dichloromethanes have been reported (Chen et al. 2017) and if this is the case in the current studies, these bacteria would be included in the total bacterial quantification. As qPCR approaches are limited to detection of only known groups participating in PCE degradation, broader approaches such as bacterial community or metagenomics approaches, or both (Miao et al. 2020) could further resolve the bacteria participating in degradation at this site. Costs for these types of analysis continue to fall, and more nuanced and detailed analysis of the microbial processes can be made to explain the distribution of chemical moieties detected, although as they also involve PCR-based amplification, the impact of inhibitory compounds should also be considered in this context (Sidstedt et al. 2019).

While iron can compete for electron exchange during degradation, it can also stimulate the process at low concentrations (AFCEE 2004). In this study, iron was not detected in concentrations high enough to compromise the natural degradation. In two cases, NI1607:2 and NI1609:3, sulfate was measured in high enough concentrations to compete as an electron acceptor in the degradation process (AFCEE 2004), although these two samples were correlated with degradation based on the results of CSIA and concentrations of chlorinated solvents. It is thus not clear how much sulfate is required to block degradation in this system. The groundwater chemistry from these two filter screens also had a divergent composition compared to the rest of the samples at the point bar, which in this case points towards a positive correlation with degradation, although why they correlate, since they are located at very different depths, is difficult to explain. Many cracks, both diagonal and vertical, were observed within the very compact silty till when investigating the cores obtained during Sonic drilling. In combination with the higher groundwater pressure in the bottom till relative to that in the top till, transport could be enabled through these fracture systems and initiate communication between the filters. Since the chlorinated solvents are dense, the free phase would not be part of this transport system. Future analyses to determine the age of the groundwater could possibly corroborate this hypothesis, explain the chemistry, and provide evidence of the communication.

No obvious trends could be observed when comparing the CSIA and microbial data, likely due to the hydrogeological complexity of the system (Fig. 5). The original isotopic composition of the contaminant might vary over time since the leakage happened over a long period and when combined with continuous transport by the groundwater flow could potentially cause non-trivial patterns in the *δ*^13^C of PCE at the site. Fluctuations in the groundwater table depth, the corresponding variation in nutrient concentrations, and preferential flow paths would exacerbate the complexity of the spatial relation between isotope signature and microbial density. The lack of a correlation between isotope and microbial data thus likely points to the time lag (and therefore spatial separation) between the historical microbial activity and the resulting isotope signal that is observed in the present. A fuller hydrogeological model could potentially help to link the time-integrated isotope signals with active microbial clusters.

The geological setting of the area combined with the results from the other analytical methods used in this study increases confidence in the interpretation of DCIP data. The *α* and *γ* anomalies are interpreted as arising from degradation and bacteria, in combination with formed gas and co-products including TCE, DCE, VC, and chloride ions (Fig. 4). The *β*-anomaly is interpreted to only belong to the geological change since this correlates well with the anomaly. The *δ*-anomaly strengthens this interpretation since it does not show a high chargeability, which should have been the case compared to the relation of *α* and *γ*. The *α*-anomaly is possibly also a reflection of the geological boundary between the two units, such as observed for the *β*-anomaly, as they are both located in the unit transition and it is an expected response of the geological change.

**Fig. 6:**
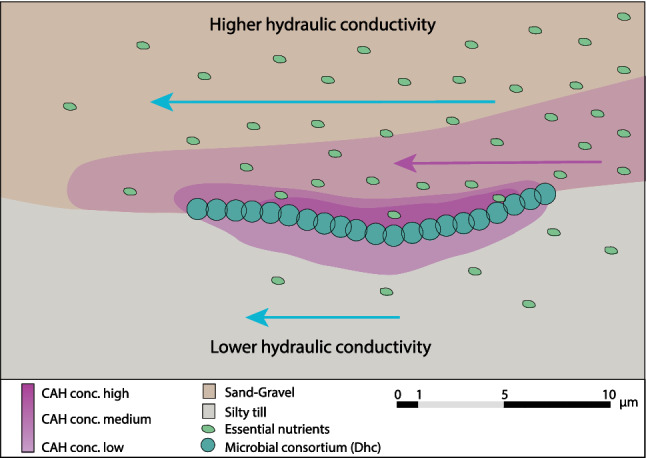
Conceptual model of the circumstances in the interface where the hydraulic conductivity changes from higher and lower. Biofilm formation by *Dehalococcoides* and the overall bacterial consortium is facilitated by the fine grained larger surface unit in areas where small contaminant pools have been formed. The sand unit above, with higher hydraulic conductivity, can benefit the bacterial consortium by providing other substances needed

The impact of the geological setting did affect where degradation occurred, in the interface between the sand unit and the top till (e.g., NI1607:2 and NI1609:2). Previous lab experiments by Sale et al. (2013) showed that lower permeability assisted microbial degradation of chlorinated solvents. Several studies (e.g., Chapman and Parker 2005; O’Hara et al. 2000) have shown that the aquitard can feed the system for years with chlorinated solvents via back diffusion. This could contribute to creating beneficial conditions at the interface between units or transition unit for microbial life based on biodegradation of the contaminants. Bacteria rarely survive on single nutrients, and the higher groundwater flow above this interface would also provide the microbial consortium with other nutrients needed for efficient degradation. When the geological information is added to the interpretation, the interface between the sand unit and the till seems to provide the optimal conditions for biodegradation of chlorinated solvents. Bacteria in nature exist largely in biofilms, a strategy that permits increased tolerance to environmental variations and interspecies cooperation that could facilitate degradation of complex molecules for nutrition (Writer et al. 2011). The compact silty till constitutes a large surface due to the smaller grain size and this could greatly enhance opportunities for *Dehalococcoides* and other bacteria to live as a consortium in biofilm while also benefiting these microbes by collecting the contaminant. The difference in hydraulic conductivity between the units can give an extra dimension to the degradation potential, as the higher flux of groundwater within the sand unit can support the bacterial consortium that has established in the transitions zone with essential nutrients, including electron acceptors, whereas the energy is taken from the degradation of contaminants (Blázquez-Pallí et al. 2019). Fluctuations due to the level of the groundwater could also introduce alternating aerobic and anaerobic conditions, which could stimulate biofilm formation and are also thought to facilitate degradation of chlorinated compounds (Richards et al. 2019). Figure 6 presents a conceptual model of the system described.

Due to the high volatility of vinyl chloride and the problems with sampling the gas phase, it is possible that vinyl chloride exists but was not detected in some of the samples and it can be debated if PCE has been completely degraded. All samples were taken in the same way, and thereby, when vinyl chloride has been detected the true concentration might have been higher.

## Conclusions

The outcome of a multidisciplinary project is a deeper understanding of the system. A natural system is complex and there is a need for different and complementary information gained by different methods. The methods in this study have helped in the interpretation and explanation of results from one method that appeared inconsistent or unclear. The combination of methods thus produces both a more nuanced and more robust interpretation of the system when more than one method can indicate the same feature or phenomena that would otherwise remain more speculative. As the methods differ in investigation scale, from microbe to geological features, and the process they observe, the combination can portray the system in various spaces and times. In this study, it was possible to make conceptual models at different scales to understand important processes active in the interface. Geological heterogeneity is important to understand and include in the interpretation of the results from a contaminated site, although more knowledge about how the response in DCIP correlates to microbial activity is needed, to answer what are the causes of different responses. Additional investigations at this site, including more sampling points, additional and diverse DCIP measurements, along with detailed descriptions of the microbial communities, would support additional analysis of the data and strengthen the conclusions presented here. In addition, by combining one or several of the methods that are deemed most informative, or by leveraging the information gained by synergies between the methods, significant cost savings are possible. This is due both to the potential for improved monitoring of remediation, as well as the ability to evaluate the effectiveness of different remediation interventions in the context of greater understanding of the subsurface processes at different scales.

More specifically we can conclude the following:

- The relationship between the degradation of the chlorinated solvents, CSIA, and microbes is complicated at this site. This is most likely due to the hydrogeological complexity and flows paths in the system.
- The specific concentrations of *D. mccartyi* for the studied site are lower than in other studies, (where pure cultures are used either in lab or injected into the ground at the site) making it difficult to detect them with the chosen qPCR assay. A microbial community likely performs degradation, and the use of other methods to capture this diversity is required.
- The geological setting, with changes in grain size between units, and in hydraulic conductivity, promotes degradation and this finding could inform design of remediation actions, i.e., including ground stabilizers with injections to lowering the hydraulic conductivity.

The following additional measurements would have answered more questions regarding the subsurface system and should be considered for additional investigation or similar studies:

- Measurements of gas concentrations in the groundwater (ethene, ethane, and methane) to evaluate the microbial activity.
- Investigation of the hydraulic conductivity to evaluate the permeability of different units and understand how they interact with each other.
- Groundwater dating with trace elements, to resolve residence time and flow paths.
- Repetition of the geophysical measurements for a time-lapse study, in order to limit the static parts (i.e., geology) of the system.

## Electronic supplementary material

Below is the link to the electronic supplementary material.

Supplementary material 1 (PDF 1216 kb)

## References

[CR1] Abdel Aal GZ, Slater LD, Atekwana EA (2006). Induced-polarization measurements on unconsolidated sediments from a site of active hydrocarbon biodegradation. Geophysics.

[CR2] Abdel Aal GZ, Atekwana EA, Rossbach S, Werkema DD (2010). Sensitivity of geoelectrical measurements to the presence of bacteria in porous media. Journal of Geophysical Research.

[CR3] AFCEE (2004). Principles and practices of enhanced anaerobic biodegradation of chlorinated solvents.

[CR4] Ajo-Franklin JB, Geller JT, Harris JM (2006). A survey of geophysical properties of chlorinated DANPLs. Journal of Applied Geophysics.

[CR5] Atekwana EA, Slater LD (2009). Biogeophysics: A new frontier in earth science research. Reviews of Geophysics.

[CR6] Auken E, Doetsch J, Fiandaca G, Vest Christiansen A, Gazoty A, Cahill AG, Jakobsen R (2014). Imaging subsurfacemigration of dissolved CO_2_ in a shallow aquifer using 3-D time-lapse electrical resistivity tomography. Journal of Applied Geophysics.

[CR7] Blázquez-Pallí N, Rosell M, Varias J, Bosch M, Soler A, Vincent T, Marco-Urrea E (2019). Multi-method assessment of the intrinsic biodegradation potential of an aquifer contaminated with chlorinated ethenes at an industrial area in Barcelona (Spain). Environmental Pollution.

[CR8] Cardarelli E, Di Filippo G (2009). Electrical resistivity and induced polarization tomography in identifying the plume of chlorinated hydrocarbons in sedimentary formation: a case study in Rho (Milan – Italy). Waste Management and Research.

[CR9] Chambers JE, Wilkinson PB, Wealthall GP, Loke MH, Dearden R, Wilson R, Allen D, Ogilvy RD (2010). Hydrogeophysical imaging of deposit heterogeneity and groundwater chemistry changes during DNAPL source zone bioremediation. Journal of Contaminant Hydrology.

[CR10] Chapman SW, Parker BL (2005). Plume persistence due to aquitard back diffusion following dense nonaqueous phase liquid source removal or isolation. Water Resources Research.

[CR11] Chen G, Kleindienst S, Griffiths DR, Mack EE, Seger ES, Löffler FE (2017). Mutualistic interaction between dichloromethane-and chloromethane-degrading bacteria in an anaerobic mixed culture. Environmental Microbiology.

[CR12] Clark K, Taggart DM, Baldwin BR, Ritalahti KM, Murdoch RW, Hatt JK, Löffler FE (2018). Normalized Quantitative PCR measurements as predictors for ethene formation at sites impacted with chlorinated ethenes. Environmental Science and Technology.

[CR13] Coplen TB (2011). Guidelines and recommended terms for expression of stable-isotope-ratio and gas-ratio measurement results. Rapid Communications in Mass Spectrometry.

[CR14] Damgaard I, Bjerg PL, Bælumb J, Scheutza C, Hunkeler D, Jacobsen CS, Tuxen N, Broholm MM (2013). Identification of chlorinated solvents degradation zones in clay till by high resolution chemical, microbial and compound specific isotope analysis. Journal of Contaminant Hydrology.

[CR15] Davis CA, Atekwana E, Atekwana E, Slater LD, Rossbach S, Mormile MR (2006). Microbial growth and biofilm formation in geologic media is detected with complex conductivity measurements. Geophysical Research Letters.

[CR55] De Wang ZPRD, Laune WHP, Masscheleyn PH (1993). Soil redox and pH effects on methane production in a flooded rice soil. Soil Science Society of America Journal.

[CR16] Elsner M, Zwank L, Hunkler D, Schwarzenbach RP (2005). A new concept linking observable stable isotope fractionation to transformation pathways of organic pollutants. Environmental Science and Technology.

[CR17] Fiandaca G, Auken E, Vest Christiansen A, Gazoty A (2012). Time-domain-induced polarization: Full-decay forward modeling and 1D laterally constrained inversion of Cole-Cole parameters. Geophysics.

[CR18] Fletcher KE, Nijenhuis I, Richnow H-H, Löffler FE (2011). Stable carbon isotope enrichment factors for cis-1,2-dichloroethene and vinyl chloride reductive dechlorination by dehalococcoides. Environmental Science and Technology.

[CR19] He J, Ritalahti KM, Aiello MR, Löffler FE (2003). Complete detoxification of vinyl chloride by an anaerobic enrichment culture and identification of the reductively dechlorinating population as a Dehalococcoides species. Applied Environmental Microbiology.

[CR20] Huang L, Sturchio NC, Abrajano T, Heraty LJ, Holt BD (1999). Carbon and chlorine isotope fractionation of chlorinated aliphatic hydrocarbons by evaporation. Organic Geochemistry.

[CR21] Hunkler, D., R.U. Meckenstock, B. Sherwood Lollar, T.C. Schmidt, and J.T. Wilson. 2008. A Guide for Assessing Biodegradation and Source Identification of Organic Ground Water Contaminants using Compound Specific Isotope Analysis (CSIA). EPA 600/R-08/148. 82 pp.

[CR22] Hunkler D, Aravena R, Butler BJ (1999). Monitoring microbial dechlorination of tetrachloroethene (PCE) in groundwater using compound-specific stable carbon isotope ratios: Microcosm and field studies. Environmental Science and Technology.

[CR23] IARC (International Agency for Research on Cancer). 2014. Trichloroethylene, Tetrachloroethylene, and Some Other Chlorinated Agents. IARC Monographs on the Evaluation of Carcinogenic Risk to Humans, Volume 106. Lyon, France. 525 pp. PMC4781308 26214861

[CR24] Jansson, R. 2018. Multidisciplinary perspectives on a natural attenuation zone in a PCE-contaminated aquifer - A case study from Hagfors, Sweden. MSc Thesis. Lund, Sweden: Lund University.

[CR25] Johansson S, Fiandaca G, Dahlin T (2015). Influence of non-aqueous phase liquid configuration on induced polarization parameters: Conceptual models applied to a time-domain field case study. Journal of Applied Geophysics.

[CR26] Johansson S, Sparrenbom C, Fiandaca G, Lindskog A, Olsson P-I, Dahlin T, Rosqvist H (2016). Investigations of a Cretaceous limestone with spectral induced polarization and scanning electron microscopy. Geophysical Journal International.

[CR27] Johansson S, Rossi M, Hall S, Sparrenbom CJ, Hagerberg D, Tudisco E, Rosqvist H, Dahlin T (2019). Combining spectral induced polarization with x-ray tomography to investigate the importance of DNAPL geometry in sand samples. Geophysics.

[CR28] Kessouri P, Furman A, Huisman JA, Martin T, Mellage A, Ntarlagiannis D, Bücker M, Ehosioke S, Fernandez P, Flores-Orozco A, Kemna A, Nguyen F, Pilawski T, Saneiyan S, Schmutz M, Schwartz N, Weigand M, Wu Y, Zhang C, Placencia-Gomez E (2019). Induced polarization applied to biogeophysics: recent advances and future prospects. Near Surface Geophysics.

[CR29] Kuder T, van Breukelen BM, Vanderford M, Philp P (2013). 3D-CSIA: Carbon, chlorine, and hydrogen isotope fractionation in Transformation of TCE to Ethene by a Dehalococcoides Culture. Environmental Science and Technology.

[CR30] Larsson, N., A.G. Christensen, H.E. Steffensen, G. Lilbæk, Y. Sandberg, F. Nilsson, B.A. Hunner, K. Baden, et al. 2017. Hagforstvätten: Data report of investigations Dec. 2013-Jan. 2017. NIRAS Sweden AB, Malmö (in Swedish).

[CR31] Lu X, Wilson JT, Kampbell DH (2006). Relationship between geochemical parameters and the occurrence of Dehalococcoides DNA in contaminated aquifers. Water Resources Research.

[CR32] Matturro B, Heavner GL, Richardson RE, Rossetti S (2013). Quantitative estimation of *Dehalococcoides mccartyi* at laboratory and field scale: Comparative study between CARD-FISH and Real Time PCR. Journal of Microbiological Methods.

[CR33] Miao Y, Johnson NW, Phan T, Heck K, Gedalanga PB, Zheng X, Adamson D, Newell C, Wong MS, Mahendra S (2020). Monitoring, assessment, and prediction of microbial shifts in coupled catalysis and biodegradation of 1, 4-dioxane and co-contaminants. Water Research.

[CR34] Munro JE, Kimyon Ö, Rich DJ, Koenig J, Tang S, Low A, Lee M, Manefield M (2017). Co-occurrence of genes for aerobic and anaerobic biodegradation of dichloroethane in organochlorine-contaminated groundwater. FEMS Microbiology Ecology.

[CR35] Nadkarni MA, Martin FE, Jacques NA, Hunter N (2002). Determination of bacterial load by real-time PCR using a broad-range (universal) probe and primers set. Microbiology.

[CR36] Nilsen J (2013). *Hagforstvätten, Main study*.

[CR37] Nivorlis A, Dahlin T, Rossi M, Höglund N, Sparrenbom C (2019). Multidisciplinary characterization of chlorinated solvents contamination and in situ remediation with the use of the Direct Current resistivity and time-domain Induced Polarization tomography. Geosciences.

[CR38] O’Hara SK, Parker BL, Jørgensen PR, Cherry JA (2000). Trichloroethene DNAPL flow and mass distribution in naturally fractured clay: Evidence of aperture variability. Water Resources Research.

[CR39] Olsson P-I, Dahlin T, Fiandaca G, Auken E (2015). Measuring time-domain spectral induced polarization in the on-time: decreasing acquisition time and increasing signal-to-noise ratio. Journal of Applied Geophysics.

[CR40] Palacky GJ (1987). Resistivity characteristics of geologic target. Geosciences Journal.

[CR41] Pankow JF, Cherry JA (1996). Dense chlorinated solvents and other DNAPLs in groundwater: History, behavior, and remediation.

[CR42] Power C, Gerhard JI, Karaoulis M, Tsourlos P, Giannopoulos A (2014). Evaluating fourdimensional time-lapse electrical resistivity tomography for monitoring DNAPL source zone remediation. Journal of Contaminant Hydrology.

[CR43] Richards PM, Liang Y, Johnson RL, Mattes TE (2019). Cryogenic soil coring reveals coexistence of aerobic and anaerobic vinyl chloride degrading bacteria in a chlorinated ethene contaminated aquifer. Water Research.

[CR44] Rosqvist H, Leroux V, Dahlin T, Svensson M, Lindsjö M, Månsson C-H, Johansson S (2011). Mapping landfill gas migration using resistivity monitoring. Waste Resources Management.

[CR45] Sale, T., B.L. Parker, C.J. Newell, and J.F. Devlin. 2013. State of the Science Review: Management of Contaminants Stored in Low Permeability Zones. Strategic Environmental Research and Development Program (SERDP) Project ER-1740.

[CR46] SEPA (Swedish Environmental Protection Agency) (2014). Nationell plan För fördelning av statliga bidrag för efterbehandling, Report 6617.

[CR47] SEPA (Swedish Environmental Protection Agency). 2015: Distributions of risk categories – divided by industry (in Swedish). Retrieved 16 March, 2017, from https://www.naturvardsverket.se/upload/sa-mar-miljon/statistik-a-till-o/fororenade-omraden/efterbehandling-riskklassford-branschvis-2015-10-12.pdf.

[CR48] SGU (Swedish Geological Survey). 2019. Hagforstvätten (in Swedish). Retrieved 20 May, 2019, from https://www.sgu.se/samhallsplanering/fororenade-omraden/fororenade-omraden-med-statligt-ansvar/hagforstvatten/.

[CR49] Sidstedt M, Jansson L, Nilsson E, Noppa L, Forsman M, Rådström P, Hedman J (2015). Humic substances cause fluorescence inhibition in real-time polymerase chain reaction. Analytical Biochemistry.

[CR50] Sidstedt M, Steffen CR, Kiesler KM, Vallone PM, Rådström P, Hedman J (2019). The impact of common PCR inhibitors on forensic MPS analysis. Forensic Science International: Genetics.

[CR51] Slater L, Lesmes DP (2002). Electrical-hydraulic relationships observed for unconsolidated sediments. Water Resources Research.

[CR52] Smith CJ, Osborn AM (2009). Advantages and limitations of quantitative PCR (Q-PCR)-based approaches in microbial ecology. FEMS Microbiology Ecology.

[CR53] Sparrenbom CJ, Akesson S, Johansson S, Hagerberg D, Dahlin T (2017). Investigation of chlorinated solvent pollution with resistivity and induced polarization. Science of the Total Environment.

[CR54] Stroo HF, Leeson A, Ward CH (2013). *Bioaugmentation for Groundwater Remediation*.

[CR56] Wiegert C, Mandalakis M, Knowles T, Polymenakou PN, Aeppli C, Macháčková J, Holmstrand H, Evershed RP (2013). Carbon and chlorine isotope fractionation during microbial degradation of tetra- and trichloroethene. Environmental Science and Technology.

[CR57] Writer JH, Barber LB, Ryan JN, Bradley PM (2011). Biodegradation and attenuation of steroidal hormones and alkylphenols by stream biofilms and sediments. Environmental Science and Technology.

