# Supplementary material for: Characterizing natural degradation of tetrachloroethene (PCE) using a multidisciplinary approach
Source: Ambio. 2020 Dec 2;50(5):1074–88. doi: 10.1007/s13280-020-01418-5 (PMC8035386; doi:10.1007/s13280-020-01418-5)
Supplement: Supplementary file 1 — Supplementary material 1 (PDF 1216 kb) [file 13280_2020_1418_MOESM1_ESM.pdf]

Ambio

Electronic Supplementary Material
This supplementary material has not been peer reviewed.

Title: **Characterizing natural degradation of tetrachloroethene (PCE) using a multidisciplinary approach**

Authors: Sofia Åkesson, Charlotte J. Sparrenbom, Catherine J. Paul, Robin Jansson, Henry Holmstrand

Appendix Tab. S1. All data including field parameters, chemical analysis, filtered volume for microbial samples, and Compound-Specific Isotope Analysis. Metals marked with asterisk (*) are acid digested.

**Well**		**NI1610:1**	**NI1610:2**	**NI1611**	**NI1609:1**	**NI1609:2**	**NI1609:3**	**NI1607:1**	**NI1607:2**	**NI1607:3**	**B29**	**B19**
**FIELD PARAMETERS**												
Temp (water)	*°C*	3.7	4.1	4.8	2.9	4.1	6.1	4	4.6	5.9	7.3	5.7
Baro	*mb*	1016	1016	1014	1006	1001	996	977	980	990	990	991
Oxidation-Redox Potential	*mV*	-82.8	-120.8	-5.7	-75.5	-126.5	-229.2	45.1	-115.3	-177.9	101.4	52.3
pH		6.56	6.6	6.5	6.67	7.19	8.5	6.54	7.85	8.23	6.48	6.86
pHmV		2.2	0.3	5.8	0.8	-27.2	-97.7	8.4	-64.3	-84.2	13.2	-7.6
Dissolved O	*% sat*	0	0.1	0	55.2	78.6	0	50.9	60.7	0.4	70.6	41.2
Dissolved Oxygen	*mg/L*	0	0.01	0	7.42	10.17	0	6.46	7.57	0.05	8.34	5.07
Electrical Conductivity	*µS/cm*	350	359	296	391	449	763	338	602	220	277	340
Resistivity	*(Ohms,cm)*	4807	4629	5494	4424	3703	2049	4926	2717	7142	5434	4651
Total Dissolved Solids	*mg/L*	227	233	192	254	291	495	219	391	143	180	221
Salinity	*ppt*	0.11	0.11	0.09	0.12	0.14	0.32	0.11	0.25	0.07	0.09	0.11
**LAB ANALYSIS**												
**Physical & chemical properties**												
Alkalinity, HCO_3	*mg/L*	120	130	89	130	150	130	93	120	74	58	98
Colour	*mg/L Pt*	50	70	10	40	20	10	10	30	5	<5	10
Hardness. German degrees	*°dH*	5.7	6.1	4.6	6.4	7.1	5.9	4.7	4.3	3.6	3.1	8.6
Conductivity 25°C	*mS/m*	24.8	24.8	21.1	27.9	28.9	64	23	44.5	17.6	22.2	22.7
pH at 20°C		6.7	6.7	6.7	6.8	7	8.2	6.6	7.8	8	6.7	8
Turbidity FNU	*FNU*	9.4	11	23	64	79	970	38	4.1	230	0.33	590
**Anions**												
Bromide	*mg/L*	<1	<1	<1	<1	<1	<1	<1	<1	<1	<1	<1
Fluoride	*mg/L*	0.2	0.2	0.24	0.27	0.33	0.61	0.22	0.58	0.51	0.19	0.63
Chloride	*mg/L*	8.5	7.6	12	9.5	8.4	55	15	41	10	25	20
Chloride	*µg/L*	8500	7600	12000	9500	8400	55000	15000	41000	10000	25000	20000
Sulphate	*mg/L*	5	4.4	6.5	7.9	1.7	100	8.5	51	2	8.8	6.8
**Nutrients**												
Ammonium	*mg/L*	0.22	0.28	0.12	0.21	<0.02	0.28	0.1	0.17	<0.02	<0.02	<0.02
Ammoniacal nitrogen	*mg/L*	0.17	0.22	0.095	0.16	<0.25	0.22	0.08	0.13	<0.25	<0.01	<0.25
Nitrate	*mg/L*	0.62	<0.3	1.3	2.5	<0.3	<0.3	2.1	<0.3	<1.1	8	<1.1
Nitrate nitrogen	*mg/L*	0.14	<0.01	0.29	0.56	<0.01	<0.01	0.47	0.04	<0.25	1.8	<0.25
Nitrate + nitrite nitrogen	*mg/L*	0.15	<0.01	0.3	0.56	<0.01	<0.01	0.47	0.037	<0.25	1.8	<0.25
Nitrite nitrogen	*mg/L*	0.0081	<0.001	0.012	<0.045	<0.001	<0.001	<0.045	<0.001	<0.001	<0.001	<0.001
Phosphate phosphorus	*mg/L*	<0.01	0.016	<0.01	<0.01	<0.2	0.18	0.012	0.075	0.13	0.011	<0.2
**Metals in water (method: ICP/AES)**												
Iron*	*mg/L*	3.2	5.1	2.3	5.9	16	1.8	1.6	1.3	5.6	0.78	86
Potassium*	*mg/L*	2.6	2.7	2.9	4.2	4.7	5.1	3.2	4	6.5	3	27
Silicon*	*mg/L*	6.9	7.1	11	12	11	15	10	7.9	27	8.9	87
Magnesium*	*mg/L*	8.3	8.3	7.8	9.4	9.8	9	7.7	7	6.8	4.8	27

Sodium *	*mg/L*	7	6.6	8.5	10	8.8	88	11	59	9.1	20	12
Aluminium	*mg/L*	0.12	0.1	0.34	0.45	0.38	0.67	0.48	0.09	1.1	0.16	16
Iron	*mg/L*	2.9	5	0.58	4.9	16	1.1	0.68	1.2	1.9	0.29	32
Calcium	*mg/L*	28	30	21	31	35	28	22	20	16	15	37
Potassium	*mg/L*	2.4	2.5	2.6	3.2	4.2	4.1	2.5	3.5	4.2	2.5	9.6
Silicon	*mg/L*	6.7	7	7.1	10	12	15	7.1	8.1	14	5.5	35
Copper	*mg/L*	<0.02	<0.02	<0.02	<0.02	<0.02	<0.02	<0.02	<0.02	<0.02	<0.02	0.04
Magnesium	*mg/L*	8.1	8.5	7.3	9.2	9.7	8.6	7.1	6.5	5.8	4.5	15
Manganese	*mg/L*	0.46	0.57	0.44	0.44	1.6	0.25	0.39	0.72	0.38	<0.02	1.1
Sodium	*mg/L*	7	6.9	8.2	10	8.8	87	11	57	7.8	20	8.1
**Metals in water (method: ICP/MS)**												
Boron*	*µg/L*	<30	<30	<30	<30	<30	59	<150	32	<150	<30	<30
Copper*	*µg/L*	0.76	<0.5	3	1.3	<0.5	0.53	1.7	<0.5	3.4	2	47
Manganese*	*µg/L*	470	560	470	470	1700	280	380	700	420	16	2100
Boron	*µg/L*	15	16	15	23	16	58	14	35	4.5	20	11
**Organic summary methods**												
DOC	*mg/L*	5.6	5.8	4.1	4.4	4.5	1.6	3.5	4.1	2.7	1.5	18
Chemical Oxygen Demand COD-Mn	*mg/L*	4.2	4.2	2.4	3.4	4.8	1.1	2	3.1	1.4	<1	4.3
TOC	*mg/L*	5.8	6	4.1	5.1	6.6	1.8	3.7	4.2	3	1.4	21
**Halogenic solvents**												
1.1.1-trichloroethane	*µg/L*	<5.0	<5.0	<10	<10	<1.0	<1.0	<10	<5.0	<5.0	<0.1	<100
1.1.2-trichlorethane	*µg/L*	<5.0	<5.0	<10	<10	<1.0	<1.0	<10	<5.0	<5.0	<0.1	**440**
1.1-dichlorethane	*µg/L*	<5.0	<5.0	<10	<10	<1.0	<1.0	<10	<5.0	<5.0	<0.1	<100
1.1-dichlorethene	*µg/L*	<5.0	<5.0	<10	<10	<1.0	<1.0	<10	<5.0	<5.0	<0.1	**140**
1.2-dichlorethane	*µg/L*	<5.0	<5.0	<10	<10	<1.0	<1.0	<10	<5.0	<5.0	<0.1	**190**
1.2-dichlorpropane	*µg/L*	<5.0	<5.0	<10	<10	<1.0	<1.0	<10	<5.0	<5.0	<0.2	<100
cis-1.2-dichloretheen	*µg/L*	**230**	**240**	**51**	**310**	**890**	**470**	**140**	**1100**	**820**	<0.1	**290**
dichloromethane	*µg/L*	<25	<25	<50	<50	<5.0	<5.0	<50	<25	<25	<0.5	<500
tetrachloroethene	*µg/L*	**4000**	**2300**	**11000**	**6600**	**160**	**720**	**10000**	**29**	**2100**	**39**	**220000**
tetrachloromethane	*µg/L*	<5.0	<5.0	<10	<10	<1.0	<1.0	<10	<5.0	<5.0	<0.1	<100
trans-1.2-dichlorethen	*µg/L*	<5.0	<5.0	<10	<10	**2.1**	**2.8**	<10	<5.0	<5.0	<0.1	<100
trichloroethene	*µg/L*	**350**	**450**	**220**	**320**	**64**	**930**	**280**	**180**	**1900**	**0.32**	**1800**
trichloromethane	*µg/L*	<5.0	<5.0	<10	<10	<1.0	<1.0	<10	<5.0	<5.0	<0.1	<100
vinyl chloride	*µg/L*	<10	<10	<20	<20	**2**	<2	<20	**33**	<10	<0.2	<200
monochlorobenzene	*µg/L*	<5.0	<5.0	<10	<10	<1.0	<1.0	<10	<5.0	<5.0	<0.2	<100
dichlorobenzene	*µg/L*	<0.6	<0.6	<0.6	<0.6	0.9	<0.6	2.1	<0.6	<0.6	<0.6	<0.6
**MICROBIOLOGY**												
Filtered volume	*mL*	250	750	750	750	500	500	500	1750	500	1000	575
Copies per mL filtered water		183	1551	611	3168	602	63	237	1647	530	6	206
**CSIA ($^{13}C/^{12}C$)**												
PCE	δ ‰	-29.3	-28.9	-29.9	-29.5	-27.6	-27.8	-31.2	-24.0	-27.7	-29.3	-30.3
TCE	δ ‰	-32.9	-32.1	-33.7	-30.7	-29.4	-28.9	-33.2	-25.0	-31.5	n.d	-27.6
cis-DCE	δ ‰	-33.3	-33	-34.7	-30.9	-28.6	-30.7	-31.8	-27.6	-26.9	n.d	-35.3
trans-DCE	δ ‰	n.d.	n.d.	n.d.	n.d.	n.d.	n.d.	n.d.	n.d.	n.d	n.d	n.d.
VC	δ ‰	n.d.	n.d.	n.d.	n.d.	n.d.	n.d.	n.d.	n.d.	n.d	n.d	-21.2

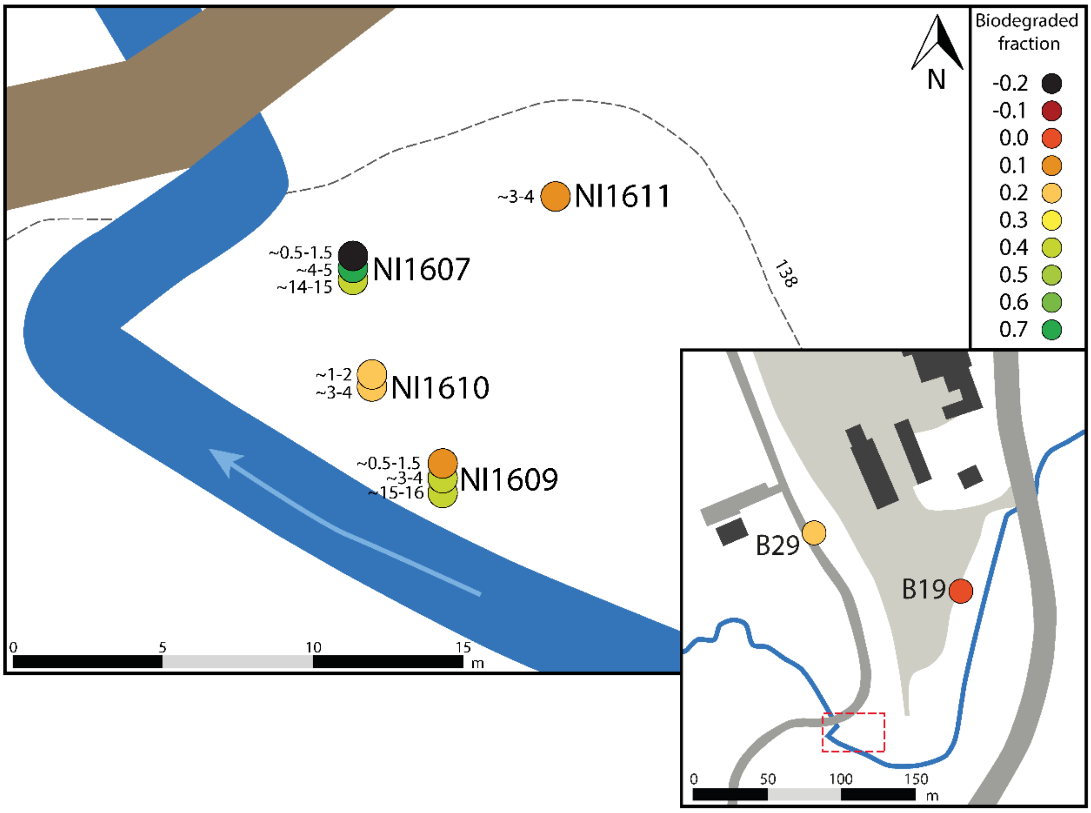

Appendix Fig. S1. Biodegradation via CSIA results of carbon. The numbers to the left correlates to the filter screen's depth, in meters below surface. Gray area marks hard surfaces, mostly macadam.

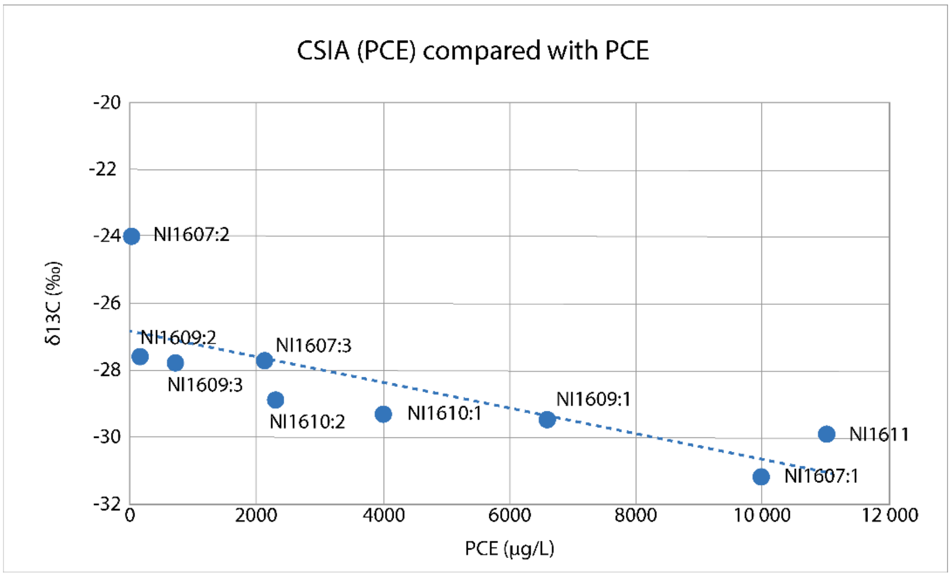

Appendix Fig. S2. Comparison between CSIA (δ13C ‰) for PCE and PCE (µg/L) shows an indication of coincide, with the exception of NI1607:2 as an outlier.

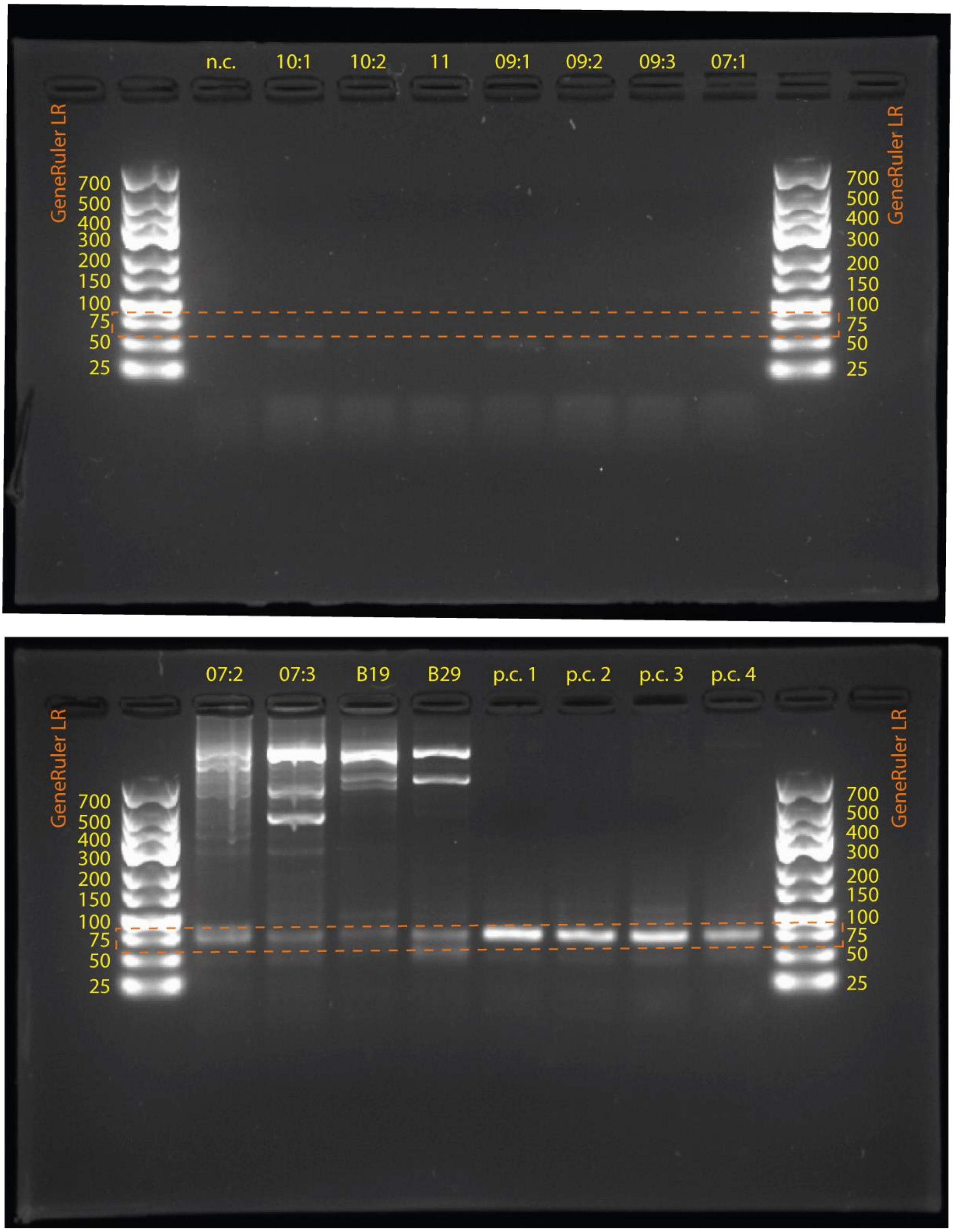

Appendix Fig. S3. Appendix Fig. S3 Gel of qPCR product (BAV1). NB! "NI16" are deleted due to space, n.c. = negative control, p.c. = positive controls in dilution serial. For the first gel are there no amplicons of the right size (71 bp), marked as an orange dashed line; however, something smaller is amplified, that is not primers dimers since they are beneath. The amplicons in a too small size can also be seen in the second gel. At the second gel are thin bands of amplicons visualized (dashed square) even thou the machine did not found amplicons of the samples. Brightest in the NI1607:2 where one out of four test showed amplification. Here are also other products amplified at ranges outside the ladder.
